# Clinical, Biochemical and Surgical Outcomes of Primary Hyperparathyroidism in the Present Era: A Prospective Study From a Tertiary Care Hospital

**DOI:** 10.7759/cureus.60965

**Published:** 2024-05-23

**Authors:** Raiz A Misgar, Munir Wani, Ajaz Qadir, Ankit Chhabra

**Affiliations:** 1 Endocrinology and Metabolism, Sher-i-Kashmir Institute of Medical Sciences, Srinagar, IND; 2 General Surgery, Sher-i-Kashmir Institute of Medical Sciences, Srinagar, IND

**Keywords:** medullary nephrocalcinosis, fractures, osteoporosis, nephrolithiasis, phpt

## Abstract

Introduction

Primary hyperparathyroidism (PHPT) has undergone a considerable change from being symptomatic to asymptomatic. This is the first large study from North India to study the clinical and biochemical features and surgical outcomes in the present era.

Study design

This is a cross-sectional study that was conducted in the Department of Endocrinology (SKIMS) from February 2021 to December 2022, in which 103 patients diagnosed with PHPT were included. Evaluation included measurement of total calcium, phosphorus, alkaline phosphate, intact parathyroid hormone, 25-hydroxy vitamin, 24-hour urinary calcium, radiological survey of hands and skull, Dual Energy X-ray absorptiometry, and ultrasonography (USG) of the abdomen. USG neck and technetium-99m sestamibi scans were used for preoperative localization; however, in cases of discordance between these investigations or suspicions of multi-glandular disease, four-dimensional computerized tomography of the neck was used. Patients were subjected to surgery according to the guidelines and monitored post-surgery for complications like hypocalcemia and hungry bone syndrome and to document the cure.

Results

The mean age of patients was 42.8±14.73 years, with a female-to-male ratio of 4.4:1. The mean eGFR of patients was 99.1±30.87 ml/min, with 55 (53.4%) of them having renal disease. Osteoporosis and fractures were present in 41 (39.8%) and 5 (4.8%) patients, respectively. Cholelithiasis and pancreatitis were present in 25 (24.3%) and 5 (4.9%) patients, respectively. Hypertension (HTN) and diabetes mellitus (DM) were the commonest comorbidities, which were present in 34 (33.1%) and 15 (14.5%) patients, respectively. Mean preoperative levels of calcium, phosphorus (PO4), alkaline phosphate (ALP), intact parathyroid hormone (iPTH), 25(OH)vitamin D, and 24-hour urinary calcium were 12.1 mg/dl, 2.35 mg/dl, 210.2 U/L, 332.9 pg/ml, 25.7 ng/ml, and 452.1 mg/day, respectively. The most common type was right inferior parathyroid adenoma, present in 45 cases (43.7%), followed by left inferior parathyroid adenoma in 31 cases (30.1%). A total of 75 patients (72.8%) underwent minimally invasive parathyroidectomy, with 68 patients (90.7%) achieving a biochemical cure. The mean adenoma weight was 3.19±2.25 g. There was no statistically significant correlation (r) between preoperative biochemical parameters and adenoma weight.

Conclusion

Despite improvements in imaging and the easy availability of immunoassays for early diagnosis, renal disease continued to be the most common presentation, followed by skeletal involvement in our population. In developing countries like India, any patient presenting with nephrolithiasis or nephrocalcinosis, low bone mass, or fragility fractures should be evaluated for PHPT.

## Introduction

Primary hyperparathyroidism (PHPT) is a disease characterized by hypercalcemia due to the excessive autonomous production of parathyroid hormone (PTH) from one or more of the parathyroid glands. After diabetes mellitus and thyroid disorders, PHPT is the third most common endocrine disorder in the West [1]. In the past, PHPT patients presented with frank symptoms and target organ damage in the form of nephrolithiasis, nephrocalcinosis, brown tumors, and fragility fractures. Nevertheless, after incorporating serum calcium measurement as a baseline parameter in the metabolic profile, the transition from symptomatic disease to asymptomatic presentation has been noted in the Western world [2]. Nowadays, a significant proportion, around 70-80%, of patients present asymptomatically, with the majority not requiring surgery and being amenable to medical intervention if necessary [3]. However, in developing countries like India and China in the past 5-10 years, most patients still present with renal and skeletal involvement [4]. Various factors contribute to the differences in terms of PHPT presentation between Western countries and India, like the lack of awareness about the disease at the primary care physician level, the limited availability of auto-analysers at primary care facilities, and prevalent vitamin D deficiency.

The present study was conducted to look for the pattern of clinical, biochemical, and surgical outcomes of PHPT in the present era in the developing country of India.

## Materials and methods

A cross-sectional study including 103 PHPT patients was carried out in the Department of Endocrinology at the Sher-i-Kashmir Institute of Medical Sciences (SKIMS) from February 2021 to December 2022 in collaboration with the Department of General Surgery. The study included PHPT patients who were diagnosed based on raised serum calcium levels above the reference laboratory limits and an increased or inappropriately normal intact parathyroid hormone (iPTH). Those with secondary or tertiary hyperparathyroidism and idiopathic hypercalciuria were excluded from the study. The objectives of this study were to investigate the clinical, biochemical, and surgical outcomes of PHPT in a cohort of patients from North India, with a focus on assessing the prevalence of symptomatic presentations, evaluating preoperative biochemical parameters, determining the efficacy of surgical interventions, and exploring correlations between biochemical markers and adenoma weight.

Sample size

Assuming a 5% significance level, the prevalence (p) of PHPT is 2% (.02), and the margin of error (e) is 4%, the sample size was calculated to be 48 by this formula: n = z2 α/2 (1-P) P = 48. To boost the power of the study by various mechanisms, like reducing sampling error, improving detection of effects, increasing test sensitivity, and enhancing generalizability, we have included all individuals who presented with primary hyperparathyroidism during the study period. Our institute also functions as a tertiary care referral centre, so a sizable number of patients from the periphery were also referred to our department.

Statistical analysis

After gathering and entering the recorded data into a Microsoft Excel spreadsheet, it was exported to the data editor of SPSS Version 20.0 (IBM Corp., Armonk, NY, USA). The categorical data were summarised as frequencies and percentages, whereas the continuous variables were reported as mean±SD. The Pearson correlation coefficient (r) was used to measure the relationship between preoperative biochemical parameters and parathyroid adenoma weight. The heatmap was prepared by incorporating the Pearson correlation coefficient (r) to determine the relationship between the mean parathyroid adenoma weight, serum calcium, phosphorus, ALP, PTH, and 25(OH)D levels with the aid of R Studio software (4.3.3). The findings of the ultrasonography (USG) neck, MIBI neck, and 4D-CT neck were compared with the gold standard histopathological examination postoperatively. As a result, parameters such as sensitivity, positive predictive value, true positive, false positive, and false negative were assessed.

Ethical clearance was obtained from the Institutional Ethical Committee in accordance with IEC/SKIMS protocol # RP 208/202. While entering the data in the Excel sheet, each patient was assigned a specific code number without mentioning their name or unique patient ID. Additionally, written informed consent was obtained through signatures or thumb impressions, and each patient was provided with a printed information sheet. A pre-designed proforma in which clinical and biochemical features, preoperative localization using various imaging methods, surgical procedure, post-operative complications, histopathology of parathyroid adenoma, and documentation of cure were incorporated for every patient. Serum total calcium, inorganic phosphorus (PO4), alkaline phosphate (ALP), intact parathyroid hormone (iPTH), 25-hydroxy Vitamin D (25-OHD), serum albumin, and 24-hour urine calcium and creatinine were among the parameters measured during the patients' evaluation. Automated techniques were used to measure serum creatinine, PO4, ALP, and total calcium. The reference range for serum calcium and phosphorus was 8.6-10.8 mg/dl and 3.5-4.5 mg/dl, respectively. Serum iPTH and 25-OHD were measured by a DXI 800 Beckman Coulter chemiluminescence random access analyzer following the manufacturer’s protocol. The normal reference range for iPTH was 12-88 pg/mL. The intra-assay and inter-assay coefficients of variation of the iPTH assay are 2.1% and 3.9%, and those of the 25-OHD assay are 3.6% and 6.5%, respectively. The glomerular filtration rate (eGFR) was calculated by the CKD-EPI creatinine equation.

Imaging methods

**Figure 1:**
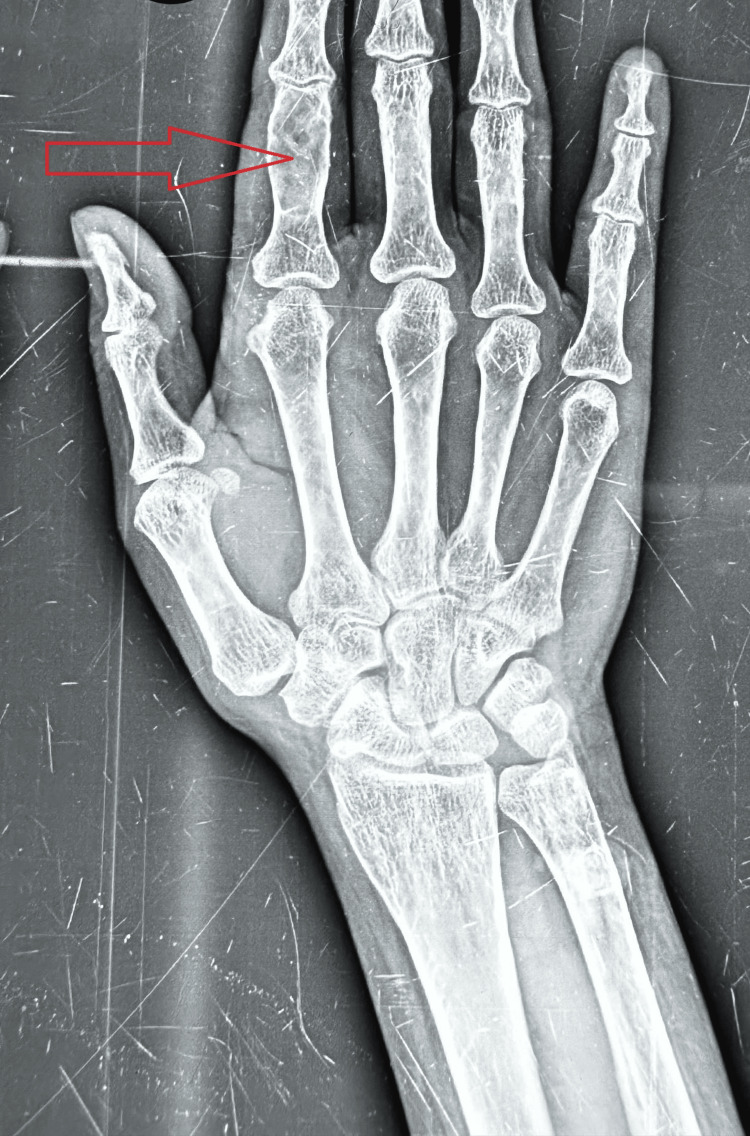
X-ray showing subperiosteal bone resorption and bone cyst in the right index finger

**Figure 2:**
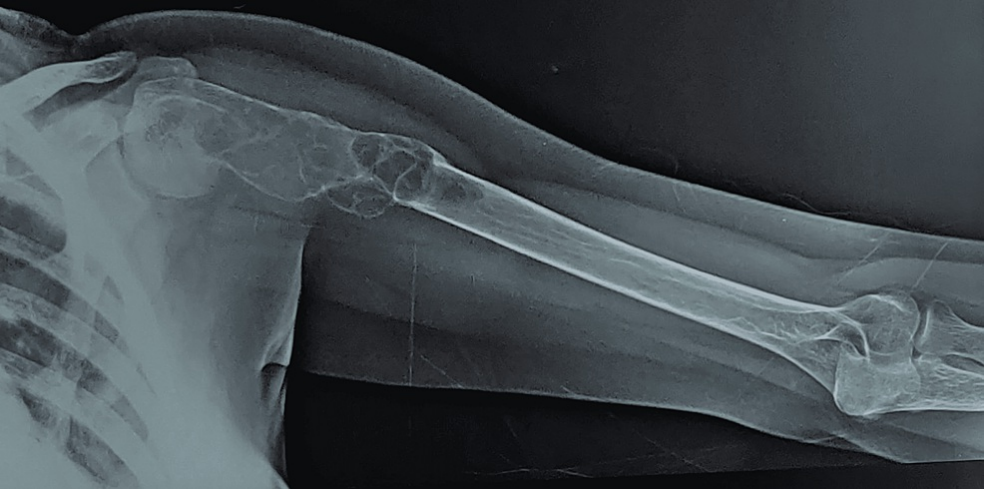
X-ray showing fracture in the left arm mimicking fibrous dysplasia

**Figure 3:**
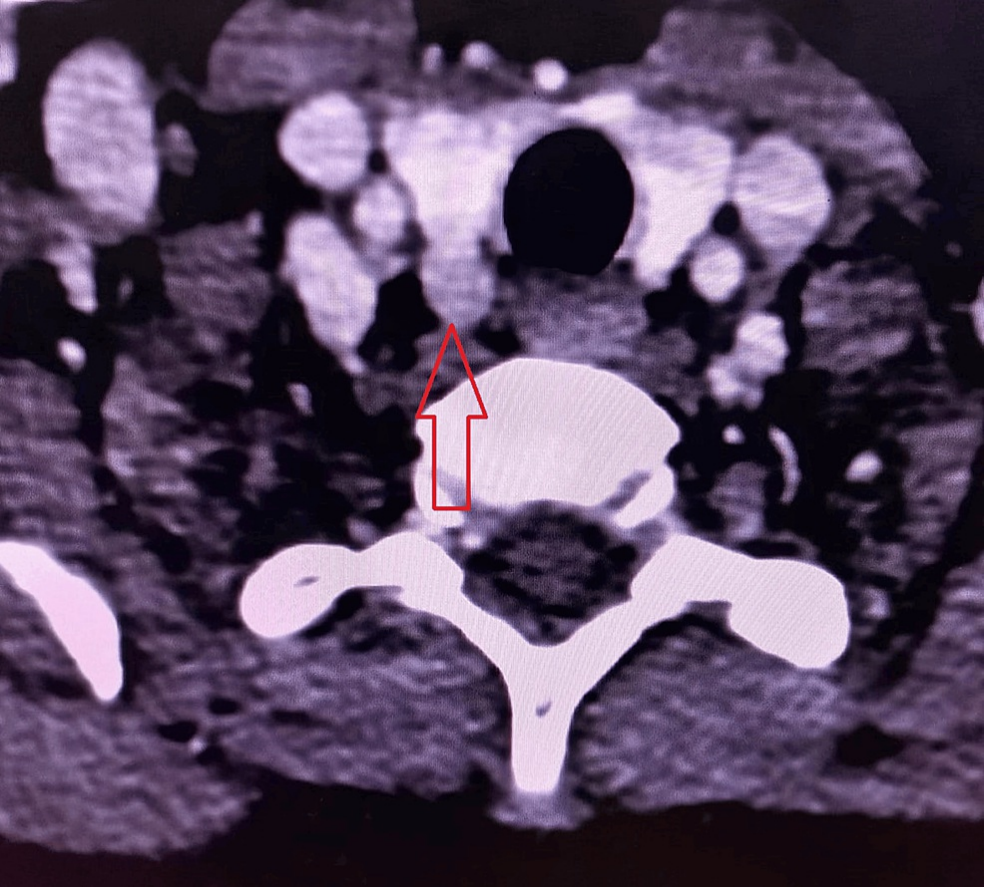
4D-CT neck showing right inferior parathyroid adenoma

A radiological survey of the hands, skull, lumbar spine, and pelvis was performed to look for subperiosteal bone resorption, brown tumors, bone cysts, and any fractures, as shown in Figure 1 and Figure 2. A brown tumor, which was defined as lytic lesions of varying shape (usually oval to round), was diagnosed on the basis of radiology findings. Three site bone mineral density (BMD) measurements, including the lumbar spine, neck of the femur, proximal femur, and distal radius, were measured with the help of lunar dual-energy X-ray absorptiometry (DEXA). Patients were reported as having osteopenia, osteoporosis, or being normal according to WHO criteria [5]. Renal ultrasonography (USG) was performed to diagnose renal stones and nephrocalcinosis. Parathyroid adenoma (PA) was localized using USG neck, technetium‑99m (Tc-99m) sestamibi (MIBI) scan, and four-dimensional-computerized tomography (4D-CT) neck as shown in Figure 3 (in the event that USG neck and MIBI results in the localization of a parathyroid adenoma are discordant). Patients were subjected to minimally invasive parathyroidectomy (MIP) according to the guidelines [6]. The removed parathyroid adenoma was weighed and histopathologically examined after surgery. Following surgery, all patients were monitored for symptoms and signs of hypocalcemia. Patients who underwent surgery were followed for six months to document cure, defined as eucalcemia persisting for six months.

## Results

In the present study of 103 PHPT patients, the female-to-male ratio was 4.4:1. The mean age of our patients was 42.8 ± 14.73 years. The most common presenting manifestation was bone pain, followed by abdominal pain and anorexia which were present in 59 (57.3%), 43 (41.7%) and 26 (25.2%) patients, respectively. Subperiosteal bone resorption and fractures were present in 25 (24.2%) and 5 (4.8%) patients, respectively. Osteoporosis was present in 41 (39.8%) patients. PHPT causes tubular dysfunction, which either results in polyuria or azotemia. It can also cause nephrolithiasis or nephrocalcinosis. In our patients, the mean eGFR was 99.1±30.87 (12.5-163.1) and nephrolithiasis and/or nephrocalcinosis were present in 55 (53.4%) patients. Cholelithiasis and pancreatitis occurred in 25 (24.3%) and 5 (4.9%) patients, respectively. Apathy and depression occurred in 24 (23.3%) and 7 (6.8%) patients, respectively. HTN and DM were present in 34 (33.1%) and 15 (14.5%) patients, respectively as shown in Table 1. The median preoperative serum calcium, PO4, ALP, and iPTH levels were 11.9 mg/dl, 2.34 mg/dl, 134 U/l and 178.4 pg/ml, respectively. The median 24-hour urinary calcium and 25(OH)D levels were 392 mg/day and 21.2 ng/ml, respectively, as shown in Table 2.

**Table 1:** Demographic and clinical features of patients (N=103)

Variables	n (%)
Gender	
Male	19 (18.4)
Female	84 (81.6)
Gastrointestinal symptoms	
Anorexia	26 (25.2)
Abdominal pain	43 (41.7)
Cholelithiasis	25 (24.3)
Pancreatitis	5 (4.9)
Psychiatric manifestations	
Apathy	24 (23.3)
Depression	7 (6.8)
Renal manifestations	
Nephrolithiasis	41 (39.8)
Nephrocalcinosis	24 (23.3)
Skeletal manifestations	
Brown tumours	3 (2.9)
Subperiosteal bone resorption	25 (24.2)
Osteoporosis	41 (39.8)
Fractures	5 (4.8)
Comorbidities	
Hypertension	34 (33.1)
Diabetes Mellitus	15 (14.5)

**Table 2:** Preoperative biochemical features of patients (N=103)

Parameters	Median	Mean ± SD
Calcium (mg/dl)	11.9	12.1 ± 1.34
PO4 (mg/dl)	2.34	2.35 ± 0.613
ALP (U/L)	134	210.2 ± 412.8
24-hour urinary calcium (mg/day)	392	452.1 ± 256.4
iPTH (pg/ml)	178.4	332.9 ± 403.6
25(OH)D (ng/ml)	21.2	25.7 ± 15.12

Preoperative localization was performed by USG neck and MIBI. However, in those patients with discordant results between USG neck and MIBI, suspected ectopic parathyroid adenoma, or those with multiglandular disease, 4D-CT neck was done. As shown in Table 3, the most common location of the adenoma was the right inferior parathyroid (45, 43.7%), followed by the left inferior parathyroid (31, 30.1%), the left superior parathyroid (5, 4.9%), and the right superior parathyroid (1, 1%). Multiglandular disease was present in 6 (5.8%) patients. The superior mediastinum (2, 1.9%), anterior mediastinum (2, 1.9%), and left carotid sheath (2, 1.9%) were the locations of ectopic parathyroid glands. The sensitivity of USG neck, MIBI and 4D-CT neck in localization of parathyroid adenoma was 88.1, 77.6% and 97% respectively as shown in Table 4.

**Table 3:** Localization of PHPT in the study population LIPA: left inferior parathyroid adenoma, LSPA: left superior parathyroid adenoma, RIPA: right inferior parathyroid adenoma, RSPA: right superior parathyroid adenoma, PHPT: primary hyperparathyroidism.

Investigations	LIPA	LSPA	RIPA	RSPA	Multiglandular disease	Not localized	Ectopic
USG neck, n (%)	31(30.1)	5(4.9)	45(43.7)	1(1)	6(5.8)	15(14.6)	---
Tc-99m MIBI, n (%)	23(22.3)	5(4.9)	36(35)	1(1)	6(5.8)	20(19.4)	4(4.8)
4D-CT neck, n (%)	10(9.7)	5(4.9)	17(16.5)	2(1.9)	5(4.9)	3(2.9)	4(3.9)

**Table 4:** Sensitivity of various investigations for localization of parathyroid adenoma PPV: Positive predictive value; TP: True positive, FP: False positive, FN: False negative

Investigations	Sensitivity	PPV	TP	FP	FN
USG Neck	88.1%	88.1%	59(78.7%)	8(10.7%)	8(10.7)
Tc-99m MIBI	77.6%	83.3%	45(67.2%)	13(19.4%)	9(13.4)
4D-CT neck	97%	91.4%	32(88.9%)	3(8.3%)	1(2.8%)

**Figure 4:**
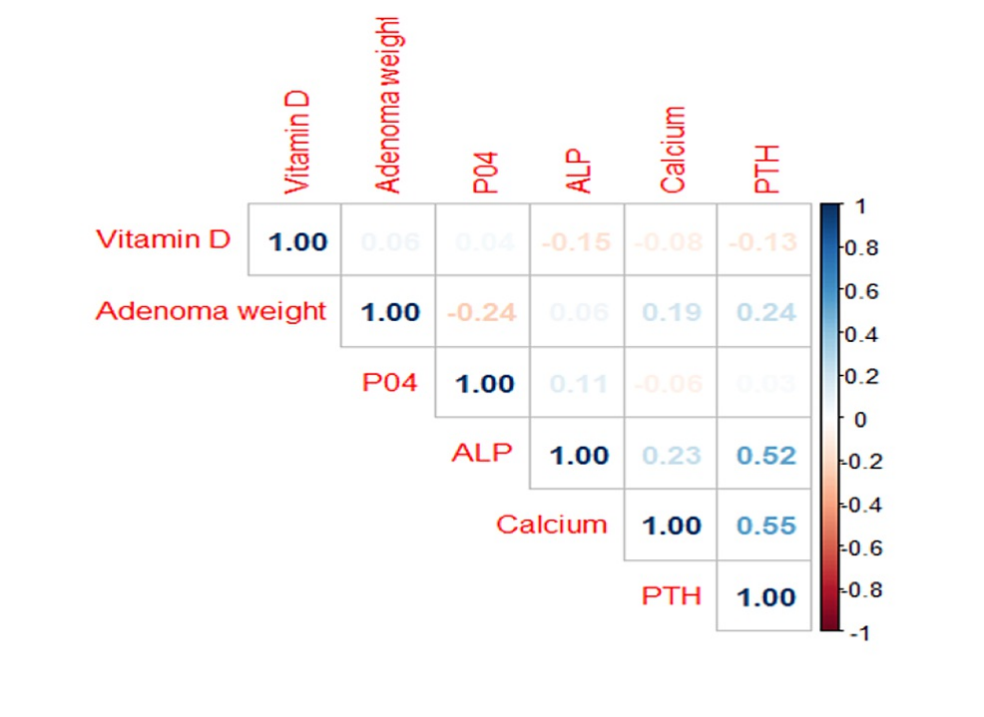
Heatmap between various preoperative biochemical parameters and adenoma weight PTH: parathyroid hormone; ALP: alkaline phosphate

In our study, 75 (72.8%) patients underwent MIP, with 68 (90.7%) achieving a cure. Seven patients had persistent disease, among them: five had suspicion of multiple endocrine neoplasia (MEN) syndrome, three had associated microprolactinomas, one had pheochromocytoma, and one had multiple renal cysts. While the remaining two patients with persistent disease were young, and had an initial imaging diagnosis of single gland adenoma, but later required second surgery owing to another gland involvement. The most common histological diagnosis was parathyroid adenoma (71, 94.5%) while the remaining 4 (6.5%) had parathyroid hyperplasia with none having parathyroid carcinoma. The mean adenoma weight was 3.19±2.25 g. As shown in Table 5, there was no statistically significant correlation (r) between preoperative biochemical parameters and mean parathyroid adenoma weight. However, using the heatmap as shown in Figure 4, there was a strong correlation between serum calcium, PTH and ALP levels. The mean postoperative serum calcium, PO4, ALP, and iPTH levels are shown in Table 6. Twenty percent of patients developed transient symptomatic hypocalcaemia within three days which required treatment. Five percent of patients developed hungry bone syndrome (HBS) which was treated with oral calcium and activated vitamin D. In our clinical practice, every PHPT patient to be operated is supplemented with oral vitamin D depending upon whether he/she is insufficient or deficient, so in our patients there was less incidence of HBS.

**Table 5:** Pearson correlation coefficient (r) between preoperative biochemical parameters and adenoma weight

Pre-operative biochemical parameters	Adenoma weight (r)	p-value
Calcium	0.114	0.34
PO4	-0.19	0.11
ALP	-0.009	0.94
iPTH	0.119	0.32
25(OH)D	0.166	0.16

**Table 6:** Postoperative biochemical profile of patients (N=75)

Parameter	Mean ± SD	Range
Calcium (mg/dl)	9.46±0.721	8.2-12
PO4 (mg/dl)	3.36±0.551	2.2-4.8
ALP (U/L)	142.1±125.9	52-902
iPTH (pg/ml)	60.7±112.2	0.5-833

## Discussion

Table 7 displays the comparison between our study and other studies. The age of PHPT presentation in India is traditionally the fourth decade [7-9]. The average age of presentation in the current study was 42 years. Two recent studies from Asia have reported a mean age of 56 and 59 years [10, 11]. Our patients continue to be much younger than patients from developed countries and other Asian countries [12]. The female predominance of PHPT is well known. In our study, an overwhelming majority of 84 (81.6%) patients were female, with a female-to-male ratio of 4.4:1. Currently, the vast majority (>80%) of patients in the USA and Europe are asymptomatic and demonstrate mild elevations in iPTH and calcium [13]. In contrast, reports from India, Thailand, and China in the past 5-10 years have concluded that most patients still manifest classical target organ complications in the form of skeleton and renal involvement [4,14]. Among the patients in our study, the majority exhibited renal and skeletal involvement. Delayed diagnosis, high prevalence of 25(OH)D deficiency, and genetic factors like calcium-sensing receptor (CaSR) gene polymorphisms could be possible explanations for symptomatic presentations of PHPT in India and other developing countries [15,16]. Renal stones (54.3%) were the most common presentation in our study. As a result of heterogeneity in the study population and the imaging techniques used to diagnose renal stones, the reported prevalence of renal stones in PHPT varies, ranging from less than 10% to more than 70% [17]. About two-thirds of patients with PHPT have previously been shown to have renal involvement (nephrolithiasis and/or nephrocalcinosis) [18]. It is unclear exactly what causes renal stones in PHPT and how they develop. It is thought that hypercalciuria is just one of the main risk factors. However, the combination of marked hypercalciuria in approximately 33% of patients with PHPT and kidney stones and clinically silent renal calculi in around 22% of asymptomatic PHPT cases underscores the need to screen people with kidney stones for PHPT, and that too in a setting like ours where symptomatic disease is still prevalent. In order to find out the incidence of stages of chronic kidney disease (CKD) in our study, we divided our patients into five groups based on eGFR as per KDIGO [19]. The mean eGFR in our study was 99.1±30.87 ml/min (12.5-163.1). A study done by Tassone et al. [20] showed a mean eGFR of 92.3±31.6 ml/min. In their study, eGFR below 60 ml/min was present in 17% of patients, while in our study, eGFR less than 60 ml/min was found in 11 (10.7%) patients.

**Table 7:** Biochemical comparison of our study versus various other studies

Total calcium (mg/dl) + SD	Serum PTH (pg/ml) + SD	Sr ALP (U/L) + SD	25(OH) Vit D (ng/ml) + SD	Mean weight of parathyroid adenoma (gram)+ SD
Our study	12.1±1.34	332.9±403.6	210.2±412.8	25.7±15.2	3.19±2.25
Shah et al. [7]	11.7±1.5	740±669.2	556.9±551.4	25±46.7	4.7±1.6
Bhadada et al. [4]	11.9±1.6	752.4±735.2	653±1180	22.9±25.1	5.6±6.5
Bandeira et al. [8]	11.9±0.2	289.7±205.4	NA	21.4±2.4	3.7±2.5

The significant morbidity associated with PHPT is mainly due to skeletal involvement. In a study conducted by Mishra et al. in 2001, involving 29 patients, it was reported that all patients had osteitis fibrosa cystica and 57% had fractures [9]. More recently, fractures were reported in 10.25% of patients and brown tumors in 6.41% of patients [18]. In the present study, subperiosteal bone resorption, fractures, and brown tumors were present in 25 (24.2%), 5 (4.8%), and 3 (2.9%) patients, respectively. These findings align with the changing pattern in PHPT presentations in India, where the prevalence of overt skeletal diseases, such as brown tumors and fractures, is decreasing. This decline may be attributed to improvements in calcium and vitamin D intake, as well as earlier diagnoses of PHPT in recent years. The average blood 25(OH)D level of 25.7 ng/ml (median 21.2) in our patients indicates an enhancement in vitamin D uptake. However, despite the relatively low prevalence of abnormalities detected in routine X-rays, bone densitometry by DEXA revealed osteoporosis in over one-third of the patients, highlighting the detrimental skeletal effects of PHPT. Two recent studies from Asia have documented osteoporosis in 32.7% and 50% of patients [10,11]. Commonly reported gastrointestinal manifestations are recurrent pain in the abdomen, constipation, heartburn, nausea, and anorexia, which occur in about 45%, 33%, 30%, 20%, and 15% of cases, respectively [21]. In our study, abdominal pain was present in 43 (41.7%) and anorexia in 26 (25.2%) patients. About 5 (5%) patients in our study had pancreatitis. An association between PHPT and pancreatitis has been suggested by several studies [22]. The highest rates of pancreatitis have been reported from India by Bhadada et al. (15%) [23] and Jacob et al. (13%) [24]. Lower rates of pancreatitis, ranging from 3.3 to 8.1%, have been observed in other studies [25]. However, compared to a population without parathyroid illness, pancreatitis appears to be ten times more common in PHPT patients. The primary cause of the disparities in pancreatitis incidence across PHPT patients is the variation in PHPT severity. The asymptomatic nature of PHPT and its early detection are reasons for the low prevalence of pancreatitis among patients with PHPT in developed nations.

With a mean serum calcium level of 12.1 mg/dl, all of our patients exhibited hypercalcemia, a finding consistent with previous studies [18,26] as well as our own. However, in contrast to our report in 2016 [18], the median serum intact parathyroid hormone (iPTH) level in our patients was 178.4 pg/mL. A recent study from China reported a median serum iPTH of 168.3 pg/mL [11]. The mean serum 25(OH)D (25.7 ng/ml) in our study was higher than that reported by previous Indian studies [27, 28]. However, vitamin D deficiency was present in 42.7% of patients (25(OH)D < 20 ng/ml) and vitamin D insufficiency (25(OH)D 20-30 ng/ml) in another 22.3% of patients. Localization of parathyroid adenoma is necessary to subject patients to MIP. USG neck was the initial investigation used, followed by MIBI for localization of parathyroid adenoma. In cases of discordant findings between the USG neck and MIBI, a 4D-CT neck was recommended. The sensitivity of the USG neck, MIBI neck, and 4D-CT neck was 88.1%, 77.6%, and 97%, respectively, for localization of parathyroid adenoma. Our study indicated higher sensitivity of USG neck in localization of parathyroid adenoma than MIBI. Various studies [29] show similar results, with the USG neck being more sensitive than the MIBI to localizing parathyroid adenoma. The positive predictive values of the USG neck, MIBI neck, and 4D-CT neck for parathyroid localization were 88.1%, 83.3%, and 91.4%, respectively, as shown in Table 3. Among the 75 (72.8%) patients who underwent MIP, 68 (90.7%) achieved a biochemical cure, while the remaining had suspicion of MEN syndrome (5 patients), and the rest were young, requiring a second surgery later on. We have previously reported a surgical cure rate of 96.5% [18]. All symptomatic patients should be considered for parathyroidectomy (PTX), as it is a safe, well-tolerated, and successful operation in the hands of experienced surgeons. However, the decision to go against the surgery could be based on some medical contraindications, prior failed surgery, or a choice. In those patients who do not meet guidelines for PTX and are not undergoing PTX, recommendations are to monitor serum calcium and 25OHD and estimate renal function (creatinine clearance, or eGFR) annually with a BMD assessment every 1-2 years [30]. The mean adenoma weight in our study was 3.19 ± 2.25 grams, which is almost consistent with the study done by Bandeira et al. [8]. However, we could not find any correlation between pre-operative biochemical parameters. The plausible reasons could be the predominantly female population in our cohort and the lesser prevalence of vitamin D deficiency.

Limitations

The study was limited by the lack of echocardiographic findings and long-term follow-up after surgery on the relapse and recurrence rates, healing of bone lesions, improvement in BMD, mortality rate, and improvement in azotemia.

## Conclusions

In developing countries like India, PHPT continues to be symptomatic. Therefore, any patient with renal stones should be suspected of having PHPT. Renal disease is the most common target organ involvement, followed by skeletal involvement. However, overt skeletal manifestations like fractures and brown tumors are declining, with osteoporosis being the predominant involvement. So, every PHPT patient should be subjected to a DXA scan. For those patients who do not meet guidelines for parathyroidectomy (PTX) and are not undergoing PTX, recommendations are to monitor serum calcium and 25OHD and estimate renal function (creatinine clearance, or eGFR) annually with a BMD (bone mineral density) assessment every 1-2 years. In patients with PHPT who are not undergoing PTX, pharmacological management should be used only for specific indications, and bisphosphonates or cinacalcet are suggested to be used for increasing BMD and lowering serum calcium levels, respectively. Daily calcium intake of 1000-1200 mg is recommended for all PHPT patients with vitamin D supplementation to maintain serum 25(OH)D levels above 30 ng/ml. In patients with suspicion of MEN syndrome, screening for anterior pituitary tumors and insulinomas should be started as early as five years of age, followed by annual screening for PHPT starting at the age of 8 years by means of serum calcium and iPTH levels.
